# Asymptomatic Dysplasia Epiphysealis Hemimelica of the Shoulder in a Skeletally Mature Patient with Normal Function

**DOI:** 10.1155/2019/5356246

**Published:** 2019-03-27

**Authors:** Vincenzo Giordano, Marcos Giordano, Carolina Giordano, José Giordano, Renato Mendonça, Hilton Augusto Koch

**Affiliations:** ^1^Serviço de Ortopedia e Traumatologia Prof. Nova Monteiro, Hospital Municipal Miguel Couto, Rio de Janeiro, Brazil; ^2^Serviço de Traumato-Ortopedia, Hospital de Força Aérea do Galeão, Rio de Janeiro, Brazil; ^3^UNIGRANRIO, Rio de Janeiro, Brazil; ^4^COFIG, Rio de Janeiro, Brazil; ^5^Serviço de Radiologia, Rede D'Or São Luiz, Rio de Janeiro, Brazil; ^6^Departamento de Radiologia, Universidade Federal do Rio de Janeiro, Rio de Janeiro, Brazil

## Abstract

Dysplasia epiphysealis hemimelica is a rare osteocartilaginous overgrowth syndrome of bone epiphysis, mostly encountered in the lower limbs of immature skeleton patients. We report a case of proximal humerus presentation in an adult male, with neither articular involvement nor clinical dysfunction. This case highlights the importance of stratification into intra- and extra-articular lesions, as this distinction ultimately influences both symptoms and treatment outcome. In addition, the case highlights the importance of specific imaging modalities, such as CT and MR images, which provide excellent anatomic location of the lesion, adequate extension of cartilaginous components, exact status of articular cartilage, and accurate assessment of neighboring structures, such as vessels, nerves, ligaments, tendons, and muscles. The imaging features are described, the relevant literature is reviewed, and salient features are discussed.

## 1. Introduction

Dysplasia epiphysealis hemimelica (DEH) is a rare disease of unknown etiology, characterized by the development of an osteocartilaginous tumor in any bone epiphysis or epiphyseal equivalent [1]. The incidence is approximately 1:1,000,000 and the male-to-female ratio is 3:1 [1, 2]. The condition was originally described in 1926 by Mouchet and Belot as “tarsomegalie” and later referred to by Trevor in 1950 as “tarso-epiphyseal aclasis” [3, 4]. Only in 1956 did Fairbank propose the current name, DEH, based on his findings that the lesion would be confined to one side of the body, either medial or lateral [5]. Azouz et al. observed different radiographic manifestations of the disease and proposed a classification system into three types, according to the phenotypic distribution: localized, affecting a single bone, classic, affecting more than one bone of a single limb, and generalized, involving an entire lower limb [6]. More recently, Clarke proposed a new classification system based on the location of the lesion, which affects clinical decision-making: intra-articular and extra-articular [2].

Although benign, DEH can have cosmetic and functional manifestations, such as clinical deformities or masses, inability to wear certain shoes, and mechanical articular impairment [1, 7, 8]. In general, intra-articular lesions tend to present more painful manifestations [2]. Imaging findings are often so characteristic that a biopsy can be averted because of the highly characteristic nature of imaging features, although it may be helpful in unusual cases, such as when the upper limbs are involved [9]. Until today, most published papers have been concerned with case reports or small case series confined to the lower limbs, mainly in skeletally immature patients [7, 8, 10, 11]. Few cases involve the upper extremity and even fewer the shoulder girdle [12–14]. In fact, the shoulder is affected in only 13.5% of all upper extremity cases reported so far [7]. We present the clinical and imaging (radiographs, CT scan, and magnetic resonance) findings of a skeletally mature patient with DEH of the proximal humerus epiphysis and normal function.

## 2. Case Presentation

**Figure 1:**
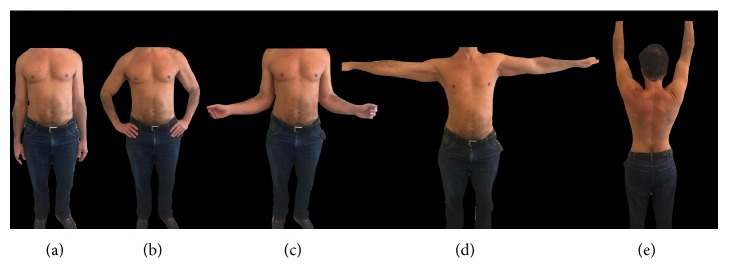
Clinical examination revealed no gross deformities or muscle atrophy on the left shoulder comparative to the contralateral upper limb. The range of motion of the left shoulder was preserved with functional shoulder symmetry. (a) Neutral position. (b) Internal rotation. (c) External rotation. (d) Abduction to 90 degrees. (e) Abduction to 180 degrees.

The patient is a 58-year-old man that presented with an insidious history of intermittent left shoulder pain, which worsens after vigorous physical training. He denied any suspicious traumatic injury over the left shoulder girdle. Clinical examination revealed no gross deformities or muscle atrophy on the left shoulder comparative to the contralateral upper limb. The range of motion of the left shoulder was well preserved with functional shoulder symmetry (Figure 1). Palpation revealed a very specific site of pain over the left acromioclavicular (AC) joint, aggravated by overhead and across-body movements. Relevant tests for degenerative rotator cuff disease and shoulder instability were negative.

**Figure 2:**
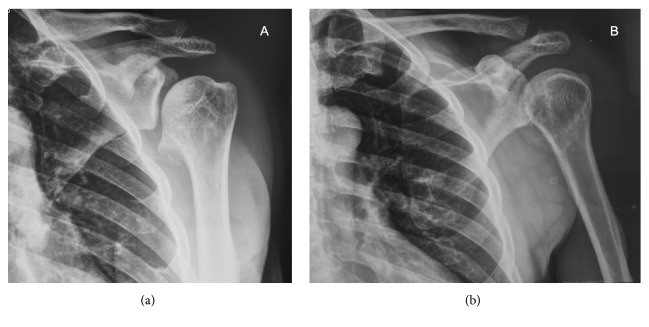
Radiographs of the left shoulder demonstrated abnormal morphology of the left humerus head, characterized by a large anteromedial exostosis arising from the epiphysis of this bone. The initial diagnosis was DEH. In addition, slight proximal migration of the distal part of the left clavicle was noted (Rockwood and Neer grade 2 AC dislocation), with no signs of AC degenerative disease. (a) True AP view and (b) AP view with maximal internal rotation.

Radiographs were obtained, demonstrating abnormal morphology of the left humerus head, characterized as an anteromedial exostosis arising from the epiphysis of this bone. There was no cortical discontinuity, periosteal reaction, calcified matrix, or articular incongruity. In addition, there were no signs of AC disease, although there was slight proximal migration of the distal part of the left clavicle, compatible with a Rockwood and Neer grade 2 AC dislocation (Figure 2).

**Figure 3:**
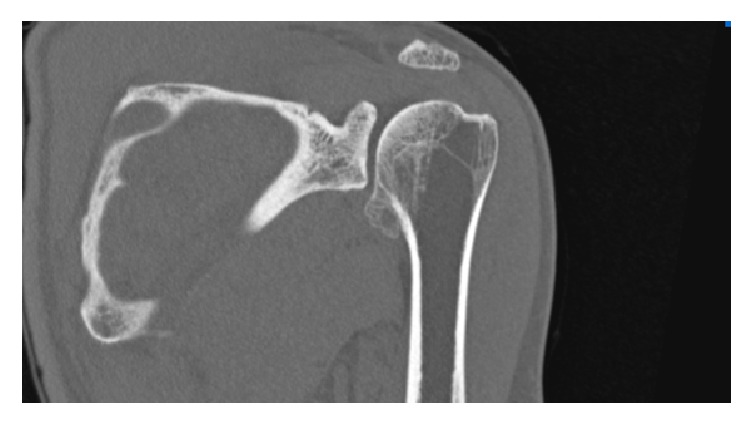
Coronal CT showed an ossified mass in the inferomedial aspect of the proximal humerus epiphysis with no articular or soft tissue involvement. The lesion was very characteristic of DEH and it was classified as extra-articular type B1 (affecting the upper limb, localized to a single bone), according to Clarke's classification system.

The initial diagnosis was DEH and he was asked multislice computed tomography (CT) and magnetic resonance (MR) images. CT showed an ossified mass in the inferomedial aspect of the proximal humerus epiphysis with no articular or soft tissue involvement (Figure 3).

**Figure 4:**
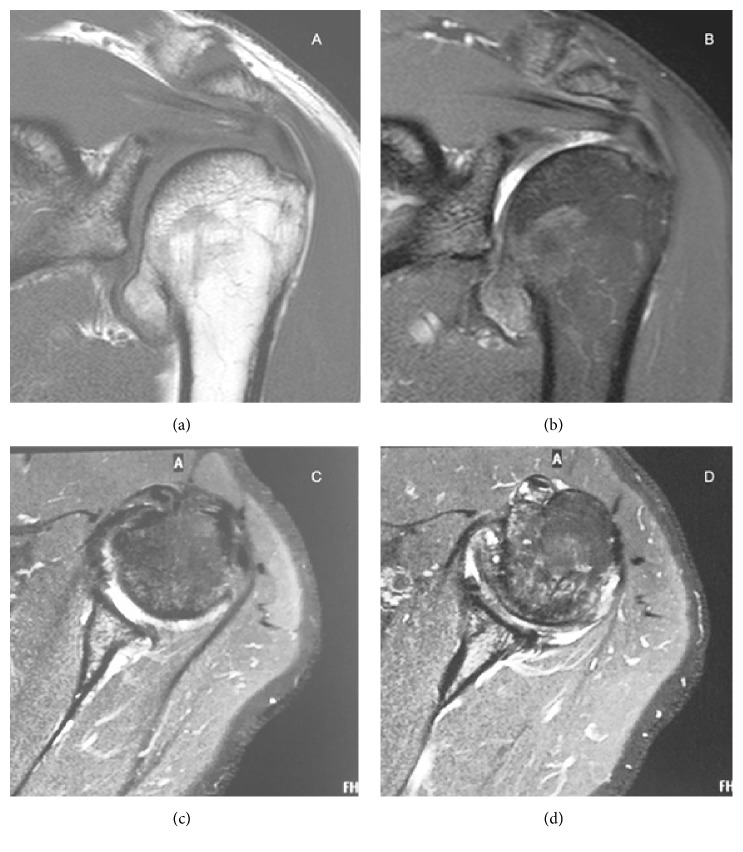
MR images of the left shoulder revealed the inferomedial mass projecting toward the inferior capsular recess, with sharpening of the articular cartilage of the humeral head, but no signs of OA. Cartilaginous components of the tumor showed intermediate T1-weigthed signal and hyperintense proton density- (PD-) weighted signal. In contrast, bone marrow of the tumor showed hyperintense T1-weighted signal and intermediate-to-low PD-weighted signal. (a) T1-weigthed coronal image, (b) PD-weighted coronal image, and (c) and (d) T2-weigthed axial images.

T1-weighted, T2-weighted, and STIR MR images of the left shoulder revealed the inferomedial mass projecting toward the inferior capsular recess, with sharpening of the articular cartilage of the humeral head, but no signs of osteoarthritis (OA). No foci of calcification or soft tissue involvement was noted (Figure 4).

The lesion was judged to be localized to the lesser tuberosity and therefore was classified as extra-articular type B1 (affecting the upper limb, localized to a single bone), according to Clarke's classification system [2]. Curiously, although MSCT and MR images were very characteristic of DEH, all radiologists' descriptions did not mention this developmental condition. Actually, according to the medical reports, the diagnosis was OA of the left shoulder.

Biopsy was not performed as imaging exams were very conclusive, showing typical alterations of DEH in the growth of the proximal humerus epiphysis. Patient was managed with analgesics and physical therapy protocol, including extracorporeal shock wave therapy (ESWT), and oriented to temporarily reduce overhead and across-body exercises. AC joint symptoms completely disappeared and he fully returned to his previous level of physical activity. Patient is periodically followed up without any recurrence of the symptoms on this shoulder.

## 3. Discussion

Although it has been described as a rare developmental disorder, recent research has greatly advanced our understanding of the pathobiology and articular involvement of DEH. Erroneously described as an osteochondroma or osteochondroma-like lesion despite its epiphyseal location, some studies demonstrated that DEH lesions differ pathologically from osteochondromas [9, 15]. In a comparative histological study, Stevens et al. found that in DEH lesions chondrocyte clusters are seen in conjunction with a thick disorganized cartilage cap and ossification centers with small amounts of unabsorbed cartilage, whereas in osteochondroma lesions cartilage is more organized and displays characteristics of the normal growth plate [15]. In addition, differently from osteochondroma lesions, collagen type II is weakly expressed and collagen type X is not detected in DEH lesions [15]. In another study, Bovée et al. observed that some gene pathways, such as EXT1 and EXT2, implicated in osteochondroma lesions, are not detected in DEH lesions [16]. Those findings have led to a hypothesis that DEH represents a primary abnormality of cartilage development that predominantly involves overexpression of resident chondroprogenitor cells within the epiphyseal cartilage, with excessive endochondral ossification during skeletal development [9].

Maturation of a DEH lesion leads to a large asymmetric overgrowth of the epiphyseal cartilage and can restrict joint motion and produce mechanical pain [1, 6–8]. The location of the lesion determines both symptoms severity and decision-making [1, 2, 7]. In general, due to potential joint incongruity, intra-articular lesions tend to be more symptomatic and sometimes can mimic conditions, such as degenerative osteoarthritis osteophytes [2]. On the other hand, the symptoms of extra-articular lesions are more nonspecific and can include sporadic pain and slightly limitation of range of motion. Our patient complained of an insidious intermittent left shoulder pain, which worsened after vigorous physical training but presented normal range of motion of the left shoulder. Palpation revealed a very specific site of pain over the left AC joint, aggravated by overhead and across-body movements. Imaging studies revealed no signs of AC disease; however, an inferomedial mass arising from the epiphysis of the proximal left humerus toward the inferior capsular recess was observed, with sharpening of the articular cartilage of the humeral head, but no signs of OA, compatible with an extra-articular type B1 DEH. Osteoarthritis of the acromioclavicular joint is a frequent cause of shoulder pain and may arise from a number of pathologic processes, including inflammatory arthritis [17]. Symptoms are often nonspecific with pain located in the neck, shoulder, and/or arm. Initial nonoperative management is aimed at relieving pain and restoring function, as occurred in our patient.

Regardless of DHE stratification into intra- or extra-articular lesion, total resection of the mass is one of the most common described interventions in immature skeletons [1, 7]. Massive ossification of the cartilaginous mass and early OA have been reported to increase over long follow-up evaluation, although few studies addressed those consequences in the adult patient [1, 7, 15]. Braman and Steward presented a case of a 16-year-old male who underwent a left shoulder custom hemiarthroplasty due to humeral head collapse after failed epiphysiodesis for continued growth of DEH at the age of 14 [12]. After two-year follow-up, prosthesis was found to be in stable position, without glenoid wear, and shoulder motion was improved but limited compared to the contralateral side. Khalsa et al. described a case of a 39-year-old female who presented with a later recurrence of DEH in the proximal tibia three years after open excision [14]. Due to its anterolateral periarticular location, authors opted for complete curettage of the lesion. After two-year follow-up, the patient remained symptom-free with no tumor recurrence. DeVine et al. reported a case of an 87-year-old female who presented with symptoms of severe degenerative osteoarthritis of her right knee due to an intra-articular lesion compatible with DEH, although described by the authors as an “intra-articular osteochondroma” [13]. She underwent a total knee arthroplasty with satisfactory final outcome.

Differently from other adult-presenting cases, our patient was found to have an extra-articular DEH lesion, with no signs of OA. It is possible that the inferomedial projection of the mass toward the inferior capsular recess but not to the glenohumeral joint reduced the risk of articular cartilage involvement, preserving joint motion and shoulder girdle function. Moreover, it is possible that rotator cuff structures were also preserved due to the inferior location of the tumor, as demonstrated by the MR images. In general, because of the natural history of DEH, it is believed that asymptomatic lesions can be observed, mainly after physis is closed, as in our patient [1, 2].

In summary, DEH is a rare osteocartilaginous overgrowth syndrome of bone epiphysis or epiphyseal equivalent, ultra-structurally and genetically distinct from osteochondroma, mostly encountered in the lower limbs of immature skeleton male patients. We report the case of a proximal humerus presentation in an adult male, with neither articular involvement nor clinical dysfunction. The case raises awareness on the stratification into intra- and extra-articular lesions, which ultimately influences both symptoms and final outcome. In addition, the case highlights the importance of specific imaging modalities, such as CT and MR images, which provide excellent anatomic location of the lesion, adequate extension of cartilaginous components, exact status of articular cartilage, and accurate assessment of neighboring structures, such as vessels, nerves, ligaments, tendons, and muscles [9, 10].
